# Evaluating feedback from an implementation advisory board to assess the rollout of a postpartum patient navigation program

**DOI:** 10.1186/s43058-024-00589-6

**Published:** 2024-05-03

**Authors:** Hannah M. Green, Brittney Williams, Laura Diaz, Viridiana Carmona-Barrera, Ka’Derricka Davis, Joe Feinglass, Michelle A. Kominiarek, Brigid M. Dolan, William A. Grobman, Lynn M. Yee

**Affiliations:** 1 Division of Maternal-Fetal Medicine, Department of Obstetrics and Gynecology, Northwestern University Feinberg School of Medicine, 250 E. Superior Street, #5-2145, Chicago, IL 60611 USA; 2 Division of General Internal Medicine, Departments of Medicine and Preventive Medicine, Northwestern University Feinberg School of Medicine, Chicago, IL 60611 USA; 3 Division of General Internal Medicine, Departments of Medicine and Medical Education, Northwestern University Feinberg School of Medicine, Chicago, IL USA; 4 Division of Maternal-Fetal Medicine, Department of Obstetrics and Gynecology, The Ohio State University, Columbus, OH 43221 USA

**Keywords:** Patient navigation, Implementation, Postpartum care, Health equity, EPIS, Qualitative research

## Abstract

**Background:**

Patient navigation is an individualized intervention to facilitate comprehensive care which has not yet been fully implemented in obstetric or postpartum care.

**Methods:**

We aimed to develop and evaluate a mechanism to incorporate feedback regarding implementation of postpartum patient navigation for low-income birthing individuals at an urban academic medical center. This study analyzed the role of an Implementation Advisory Board (IAB) in supporting an ongoing randomized trial of postpartum navigation. Over the first 24 months of the trial, the IAB included 11 rotating obstetricians, one clinic resource coordinator, one administrative leader, two obstetric nurses, one primary care physician, one social worker, and one medical assistant. Members completed serial surveys regarding program implementation, effects on patient care, and areas for improvement. Quarterly IAB meetings offered opportunities for additional feedback. Survey responses and meeting notes were analyzed using the constant comparative method and further interpreted within the Exploration, Preparation, Implementation, Sustainment (EPIS) Framework.

**Results:**

Members of the IAB returned 37 surveys and participated in five meetings over 24 months. Survey analysis revealed four themes among the inner context: reduced clinician burden, connection of care teams, communication strategies, and clinic workflow. Bridging factors included improved patient access to care, improved follow-up, and adding social context to care. Innovation factors included availability of navigators, importance of consistent communication, and adaptation over time**.** Meeting notes highlighted the importance of bidirectional feedback regarding implementation, and members expressed positive opinions regarding navigators’ effects on patient care, integration into clinic workflow, and responsiveness to feedback. IAB members initially suggested changes to improve implementation; later survey responses demonstrated successful program adaptations.

**Conclusions:**

Members of an implementation advisory board provided key insights into the implementation of postpartum patient navigation that may be useful to promote dissemination of navigation and establish avenues for the engagement of implementing partners in other innovations.

**Trial registration:**

ClinicalTrials.gov, NCT03922334. Registered April 19, 2019. The results here do not present the results of the primary trial, which is ongoing.

Contributions to the literature

- Implementation science has consistently underscored the importance of incorporating the perspectives of various partners into the implementation of novel interventions.
- The implementation of patient navigation, an emerging innovation to reduce maternal health disparities and improve outcomes, has not yet been evaluated with respect to the perspectives of clinical partners.
- Bidirectional feedback between program implementers and an implementation advisory board, both through structured surveys and scheduled meetings, may serve as a useful mechanism to optimize implementation for innovations both within the research setting and in clinical practice.

## Background

As disparities in postpartum care persist despite standardized recommendations to optimize care, [1–5] postpartum patient navigation could serve to promote the long-term health of birthing individuals. Postpartum patient navigation, an individualized intervention which identifies and addresses barriers to care, is emerging in the field of obstetrics as a method to facilitate healthcare access and promote comprehensive postpartum care [6]. Navigators’ roles include assisting patients with care coordination, addressing social determinants of health, and fostering patient activation to promote high-quality care [7]. Despite this promise, patient navigation has not yet been integrated into usual postpartum care [6, 8].

As with all emerging interventions or the application of established interventions to novel contexts, ascertaining the determinants of implementation is necessary to facilitate programmatic success. Such identification would provide valuable information for creators of new postpartum navigation programs to optimize implementation if the intervention is shown to improve postpartum health. In the implementation of any novel intervention, various collaborators, including those who work closely with implementers or individuals who receive the intervention, may have particularly important perspectives on determinants of implementation. Given that engagement of clinical and administrative partners is integral to implementation science overall, [9] identifying and incorporating these partners within postpartum navigation programs is an important component of program development and evaluation. Thus, we aimed to examine the role of implementing partners’ feedback in the context of an ongoing randomized controlled trial of postpartum patient navigation in an urban academic medical center.

## Methods

This study assessed the value of a clinical Implementation Advisory Board (IAB) in supporting an ongoing randomized controlled trial of postpartum patient navigation (NCT03922334) [10]. The trial, “Navigating New Motherhood 2 (NNM2)” aims to enroll 400 pregnant or postpartum individuals enrolled in Medicaid for obstetric care, and to randomize them to receive either 1 year of individualized postpartum patient navigation or usual care. This trial is being conducted at a large, urban academic medical center that cares for diverse birthing individuals and its primary aim is to determine whether participation in patient navigation program improves postpartum health outcomes. This analysis aims to present the findings from IAB surveys and meeting notes to identify determinants of program implementation, as perceived by the IAB, as well as assess their perspectives on program effects and integration into the clinic setting. In doing so, we aim to elucidate the potential value of an IAB for optimizing the ongoing implementation of novel innovations overall.

As part of study protocol, investigators identified implementing partners and assembled an IAB to create a formal process for clinical partners and research team members to exchange bidirectional feedback regarding the implementation and effects of the ongoing innovation. This process allowed for investigators to provide updates on research aims, while implementing partners could provide information on the real-time effects of the program, as well as make suggestions to promote integration into the clinic setting in which the program took place. Over the first two years of the trial, the IAB included 11 rotating obstetricians, one clinic resource coordinator, one administrative leader, two obstetric nurses, one primary care physician (PCP), one social worker, and one medical assistant. Research staff formally recruited IAB members, all of whom worked within the clinic site of program implementation, via direct face-to-face or electronic discussions about the program. IAB members participated voluntarily and were not compensated. Through serial surveys sent to all members every two months, the IAB provided real-time feedback to investigators and program staff regarding implementation of the intervention. While surveys were sent to all members, an initial screening question allowed respondents to terminate the survey if they had not interacted with the patient navigation program in the 2 months prior. Surveys included respondents’ identities and contained seven open-ended questions that generated responses allowing for in-depth qualitative interpretation. Surveys queried respondents’ opinions regarding program integration, modes of communication with navigators, areas for programmatic improvement, and effects of the program on clinical care (Table 1). The research team developed these questions a priori in alignment with study goals to assess implementation. This analysis uses the IAB responses to assess program implementation, and no data from the RCT are presented herein.

**Table 1:** Serial survey questions (q = 2 months) sent to IAB regarding program implementation

- Did you have any exposure to the Navigating New Motherhood 2 program since the last survey?
- Tell us how Navigating New Motherhood 2 is integrating into the clinic. What is going well and should be retained?
- What aspects of the program (scheduling, structure, integration into clinic, etc.) can be improved and should be removed or modified?
- Please provide your feedback on the navigators. Do you have comments on additional training needs, feedback on their performance, communication skills, or other areas for improvement?
- What types of communication are you using with the navigators and what ideas do you have for improving communication?
- How is the NNM2 program affecting how you provide clinical care?
- What ways can the navigators make your clinical duties more effective/manageable?

In addition to the surveys, hour-long quarterly meetings provided opportunities for additional feedback. Meetings included a presentation by the research team to IAB members about the progress of the study as well as structured discussion in which IAB members provided feedback to the research team. Additionally, the research team offered tips and education to the IAB about the patient navigation program, provided updates on programmatic evolution in response to prior feedback, and shared initial research findings from early-phase work [11, 12]. Meetings were initially in-person but converted to virtual at the start of the COVID-19 pandemic. One research team member took comprehensive notes to document all discussions at meetings.

Survey responses and IAB meeting notes were analyzed by the primary author using the constant comparative method and grounded theory to inductively identify initial themes. Given the small quantity of data, no analytic software was used, and analysis was conducted on word processing programs within a secure server. In an initial round of coding, the primary author coded survey response data and meeting notes and consolidated these codes into initial themes to identify determinants of program implementation as perceived by the implementation advisory board. This thematic analysis was discussed, refined, and finalized with the entire research team through an iterative process. The primary author then conducted a second round of analysis in which these themes were mapped onto three of four constructs (inner context, bridging factors, innovation factors) within the Exploration, Preparation, Implementation, Sustainment (EPIS) Framework, a common framework for the qualitative assessment of implementation strategies [13]. After mapping themes onto EPIS constructs, the analysis was reviewed and finalized with the entire research team to maximize reliability.

The EPIS Framework was selected primarily for its articulation of the relationships between the primary setting of the innovation and the larger context of implementation, suitable for a broad intervention such as patient navigation [14]. Multiple published studies have used the EPIS framework to primarily assess implementation, with less focus on other phases of the implementation process (i.e. exploration, preparation, and sustainment), supporting this framework as appropriate to structure our analysis of program implementation [13].

## Results

During the period of data collection, 18 individuals served on the IAB. Given the presence of a screening question which allowed individuals to terminate the survey if they had not interacted with the patient navigation program, 11 individuals provided qualitative survey data. We received 39 qualitative survey responses from these 11 unique individuals including obstetricians, a research coordinator, a clinic administration, nurses, primary care physicians, and a social worker. Among these 11 individuals, the average survey response rate was 68%. Given the survey contained only open-ended questions, there was variation in the level of detail provided in responses, though nearly all provided responses contained substantive information regarding implementation suitable for qualitative analysis. To supplement surveys, we held five meetings over 24 months with additional members of the IAB.

Survey responses revealed positive themes across the inner setting construct, defined as characteristics of the organization in which the innovation takes place. One obstetrician noted, “the only aspect I would change…is to have every one of our patients have a navigator.” Specific themes included facilitating a reduced burden of care for providers, allowing one obstetrician to “focus…more on…medical care than on…care coordination.” In addition, increased connection between obstetric, specialty, and primary care teams, successful communication strategies, and successes in clinic workflow characterized the inner setting (Table 2).

**Table 2:** Implementation Advisory Board members’ survey feedback about navigation implementation analyzed across EPIS^a^ constructs

Inner Context	
Reduced clinician burden	“I don’t feel as overwhelmed at the prospect of coordinating the many multi-faceted aspects of postpartum care for my patients…” (MD, OB/GYN)
Connection of care teams	“[Navigation] affects clinical care by increased team collaboration of patient needs” (Social Worker)
Communication strategies	“[EMR] message is best and easiest for me to get back to them quickly and efficiently” (Resource Coordinator)
Clinic workflow	“The NNM2 team has integrated rather seamlessly into our clinic…in a manner that compliments the entire practice without impeding on the excellent clinical care given” (Administrator)
**Bridging Factors**	
Improved patient access to care	“The navigator had already reached out to the patient, determined the issue that led to the no show, and helped get the patient rescheduled” (MD, Primary Care)
Improved follow-up	“[The navigator] follow[ed] up quickly…with a patient who I was concerned about their postpartum depression, helping them get into community resources” (MD, OB/GYN)
Adding social context to care	“[They] clued me into patient issues that I otherwise might not have been aware of – living situations, relational or safety issues, etc.” (MD, OB/GYN)
**Innovation Factors**	
Availability of navigators	“[Navigators are] frequently around the patients…when I needed something I could easily find them” (MD, OB/GYN)
Importance of consistent communication	“I appreciate being contacted ahead of time by the navigator…it helps me direct my clinical care” (MD, OB/GYN)
Adaptation over time	“[Navigators have been] learning quickly and adapting to new changes efficiently” (Resource Coordinator)

^a^Exploration, Preparation, Implementation, Sustainment Framework

Bridging factors, or themes related to the connection of the inner organization to broader systems, included improved patient access to care. The clinic social worker noted navigators assisted her “in identifying barriers to care” such as transportation and insurance issues. One obstetrician noted this resulted in patients being “less likely to cancel their appointments.” Likewise, improved follow-up in care among navigated patients and the incorporation of participants’ social needs into postpartum care added “so much to [one obstetrician’s] understanding of the patient’s complex social background” (Table 2).

Finally, positive innovation factors, or characteristics of the innovation itself, included a high degree of availability of navigators to providers, as one obstetrician stated “navigators are easily reached and have a presence in clinic.” Additional innovation factors included the importance of consistent communication between navigators and care teams and adaptation of the innovation over time, especially in areas for growth specifically identified by IAB feedback (Table 2). The clinic patient service representative noted navigators were “very much susceptible [i.e., accepting of] to feedback” and adapted “to the ever-changing policies [in clinic] impressively well.”

Analysis of IAB meeting notes demonstrated positive perceptions of program implementation, specifically regarding its smooth incorporation into clinic workflow and responsiveness to feedback. These meetings also provided members with the opportunity to expound on prior survey responses and provide concrete suggestions for program modifications to promote implementation. The group setting of IAB meetings allowed for brainstorming among multiple perspectives at once. In initial meetings, members suggested programmatic improvements regarding navigators’ communication strategies, specifically regarding navigators’ inconsistent use of the electronic medical record messaging system. In response to this feedback, navigators began integrating the electronic medical record into their daily tasks, contacting providers, scheduling appointments, and coordinating care through the EMR, and later data demonstrated this adaptation successfully ameliorated these issues, ultimately promoting implementation as communication “significantly improved” and had “only grown to be better.”

## Discussion

In summary, we identified that incorporation of an IAB into the implementation of a postpartum navigation program provided important insights and feedback regarding program rollout and evolution. Survey responses and meeting notes demonstrated positive assets of the innovation and its implementation, including attention to social components of care, navigator availability, and adaptable communication styles, which all were perceived to improve clinical care. A variety of implementing partners, including clinicians, administrators, and social workers, elucidated facilitators to implementation, as well as provided suggestions for improvement, allowing for program responsiveness and evolution. As navigators themselves worked closely with these partners in daily activities, this bidirectional feedback fostered adaptation of program activities.

Our results also support the overall utility of an IAB for optimizing the implementation of novel innovations. The IAB served as a real-time mechanism for adjustments to program implementation which incorporated a variety of perspectives. The bidirectional nature of feedback, in which research staff provided updates to implementing partners that were relevant to the program’s progress and adaptations while partners themselves made suggestions for programmatic improvement, was an effective way to tailor program rollout without interfering with ongoing program operations, as evidenced by improvements made directly in response to IAB comments (e.g. integration of the EMR into navigator task flow). These results support the use of a IAB for optimizing implementation of ongoing innovations.

This analysis has a few limitations to note. The lower survey response rate may introduce bias, as IAB members with more frequent interactions with navigators and strong opinions regarding implementation may have been more likely to respond. These data, however, would represent the pitfalls and assets of implementation as perceived by individuals most regularly interacting with the innovation, while IAB meetings provided an opportunity for all participants to offer opinions regarding implementation. Additionally, our method of qualitative analysis identified overarching themes in survey responses and was not conducive to identifying granular feedback and suggestions from the IAB, such as logistical modifications or identifying specific cases of missed opportunities. These suggestions, however, represent site-specific data which are less integral to our larger findings regarding the program overall and the role of the IAB in promoting its implementation. Lastly, the setting of implementation, an ongoing research study at an urban academic medical center, may impact the generalizability of our findings. Other settings, including smaller health systems not conducting research on the innovation, may not have the resources to incorporate an IAB in program rollout. Our findings, however, support the use of an IAB in implementation of postpartum navigation or other similar interventions, when feasible, as a useful mechanism for continuous feedback to streamline implementation of a novel program.

Future work may use the EPIS framework to explore the role of an IAB in program sustainment past the initial implementation phase, particularly as interventions move from the research context to long-term implementation. Furthermore, future incorporation of implementing partners into patient navigation programs or other maternal health interventions should include the perspectives of birthing people themselves and members of the communities in which they live. As we began the IAB at the beginning of our trial, no patients were available to participate, yet future work should incorporate these important perspectives.

## Conclusion

Identifying feedback regarding the implementation of our postpartum navigation program, as perceived by an IAB, may elucidate determinants of implementation that can promote its future dissemination, [15] while the emphasis on program adaptation this study found supports the use of IABs in implementation for similar programs. Findings underscore the importance of including implementing partners in the rollout of novel interventions, both when the interventions are conducted in the research context and clinical practice.

## Data Availability

Data are not publicly available given the ongoing nature of the trial and the sensitive nature of the information. Data however are available from the authors upon reasonable request and with permission of the Northwestern University IRB, upon conclusion of the ongoing trial.
